# Delayed Bowel Perforation after Routine Distal Loopogram Prior to Ileostomy Closure

**DOI:** 10.1515/med-2020-0037

**Published:** 2020-04-04

**Authors:** Keat Seong Poh, Siew Yep Hoh, Rezal Aziz, Shun Siang Chong, April Camilla Roslani

**Affiliations:** 1 Department of Surgery, University Malaya, Kuala Lumpur, Malaysia

**Keywords:** Anastomosis leak, Bowel perforation, Distal loopogram, Ileostomy, Ultralow anterior resection

## Abstract

Ultra-low anterior resection for low rectal cancer is usually done with a covering ileostomy as a safety measure to reduce the consequences of distal anastomotic failure. In many centres, distal loopogram is performed routinely, prior to the closure of the loop ileostomy, to assess the integrity of anastomosis. Distal loopogram is generally considered a safe procedure with very low complication rates, especially when water-soluble contrast is used. We report two cases of delayed bowel perforation which led to severe sepsis and generalized peritonitis after distal loopogram prior to ileostomy closure. Our cases highlight the potential dangers of distal loopogram. Therefore, the routine usage of this procedure should be scrutinized and the patient needs to be properly counselled prior to the procedure.

## 1 Introduction

Ultra-low anterior resection (ULAR) has a significant anastomotic leak rate with published rates varying from 2.8 to 25% [1]. A covering ileostomy is usually performed to decrease clinical anastomotic leak and reoperation rates. Contrast studies like distal loopogram (DL) are routinely performed in many centres to assess the integrity of the ULAR anastomosis, before closure of the covering ileostomy. However, the value of this practice is questionable [2, 3], and some use distal loopogram only selectively for patients with abnormal findings on digital rectal exam and rigid proctoscopy [4].

Distal loopogram is generally considered safe, with a very low complication rate, especially when water-soluble contrast (e.g. Gastrografin) is used. However, we recently encountered two cases of iatrogenic, delayed bowel perforations post-routine distal loopogram, which led to significant peritoneal contamination and sepsis, requiring emergency surgery.

## 2 Case reports

### 2.1 Case (1)

**Figure 1:**
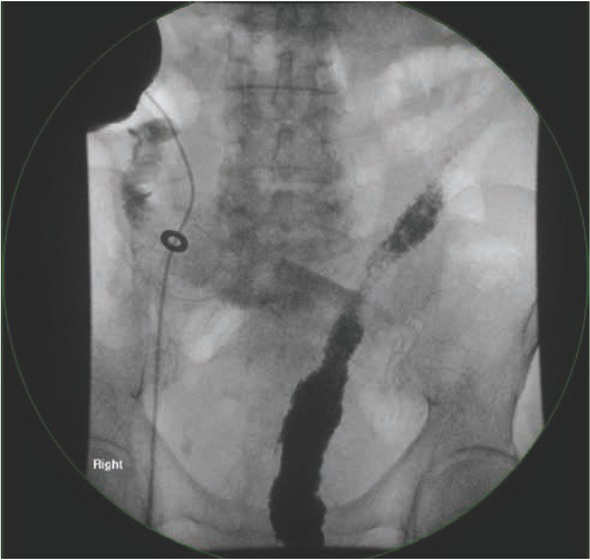
(Case 1) Routine Distal loopogram done 10 days post ULAR showing no evidence of contrast leak. Patient was discharged home well after the procedure.

**Figure 2:**
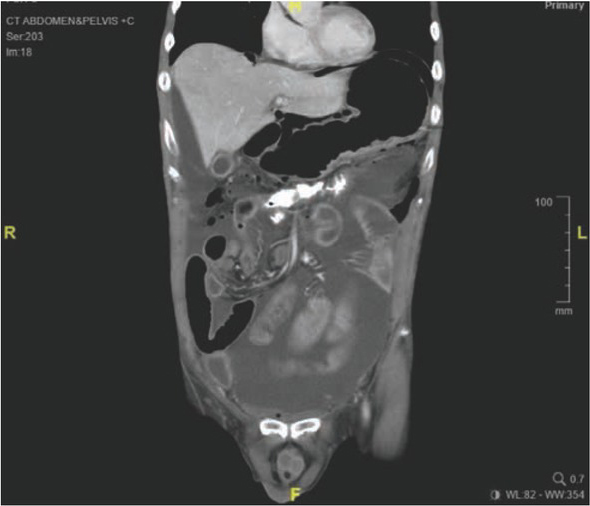
(Case 1) Two days after the distal loopogram was done, patient presented with generalised peritonitis and his CT abdomen showed gross ascites and pneumoperitoneum.

**Figure 3:**
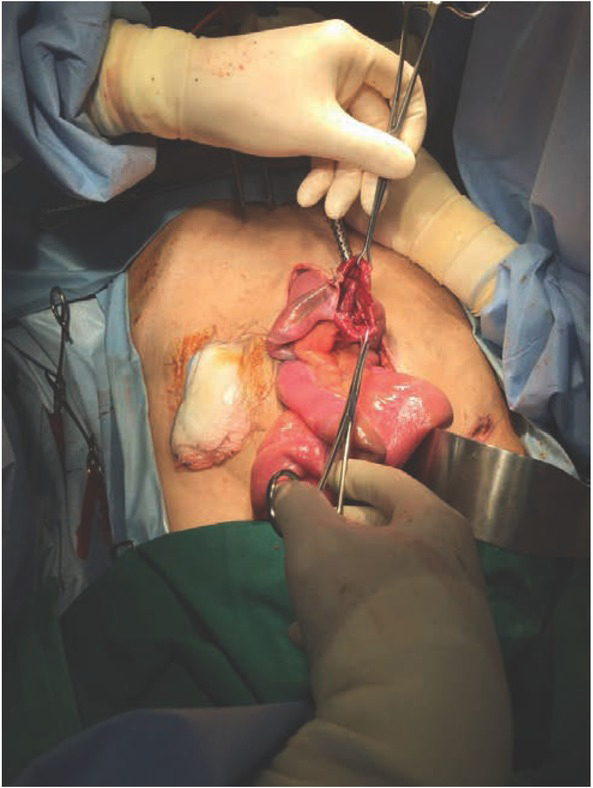
(Case 1) Intraoperatively, large perforation noted at the distal ileum, proximal to the covering ileostomy. There was generalised purulent peritonitis.

A 66-year-old elderly man, who had Stage III mid-rectal carcinoma (T3N1M0), underwent ULAR with a covering ileostomy. The patient had no other previous medical history. The operation was performed six weeks after completing neo-adjuvant concurrent chemo-radiotherapy. On the tenth post-operative day,he underwent a distal loopogram as an outpatient procedure, to assess the ULAR anastomosis. During the procedure, the insertion of the Foley’s catheter into the distal loop of the ileostomy required multiple attempts, owing to the difficulty in identifying the correct lumen. The distal loopogram showed an intact ULAR anastomosis, with no evidence of an anastomotic leak (Figure 1), thus the patient was discharged home, and scheduled for elective closure of ileostomy at a later date. However, two days after the distal loopogram, the patient returned to the hospital with septic shock (blood pressure 95/60mmHg, pulse rate 128 bpm, afebrile) and generalised peritonitis. Erect chest X-ray showed pneumoperitoneum. After adequate fluid resuscitation, CT abdomen/ pelvis was performed. CT abdomen showed leakage of contrast in the right hypochondrial region with pneumoperitoneum (Figure 2). The patient underwent emergency laparotomy the same day. Intra-operatively, there was a large small bowel perforation, 10cm proximal to the covering ileostomy (Figure 3), with generalised peritoneal contamination. The perforated segment was resected, and a double-barrelled ileostomy constructed. Post-operative recovery was complicated with pneumonia, kidney impairment, and high output stoma. He spent the next two months in the hospital but eventually recovered fully. His stoma was closed during the same admission with no further complication.

### 2.2 Case (2)

**Figure 4:**
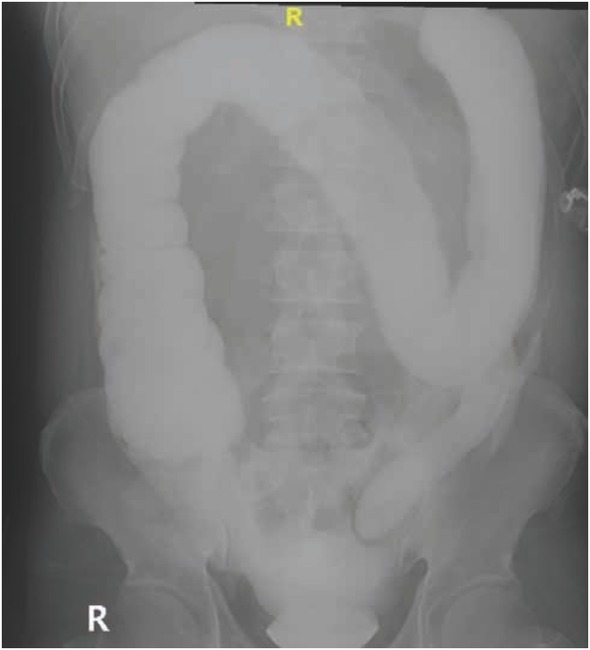
(Case 2) Plain abdominal x-ray done eight hours after the distal loopogram procedure showed extraluminal contrast.

**Figure 5:**
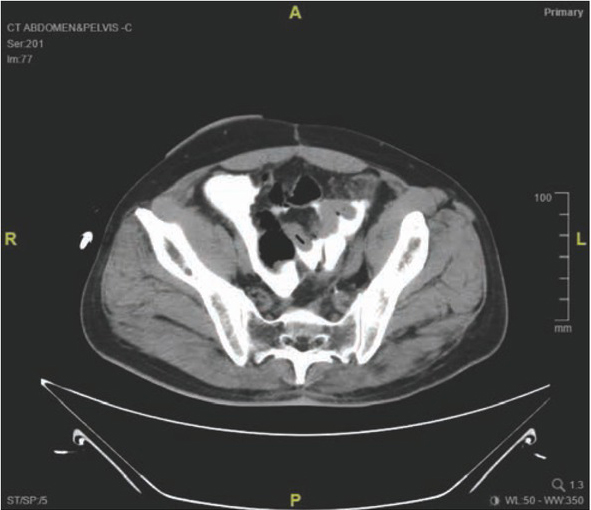
(Case 2) CT abdomen done eight hours after the distal loopogram, showed contrast pooling at the pelvis.

**Figure 6:**
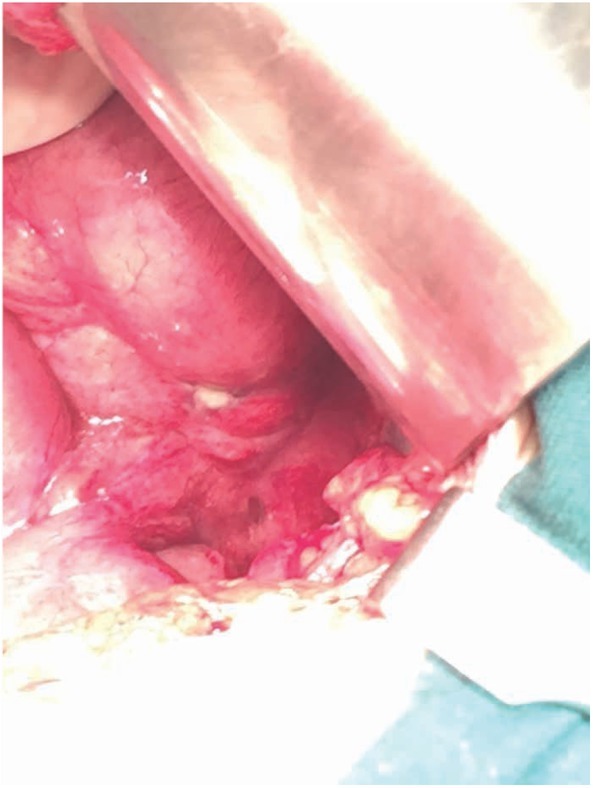
(Case 2) Intraoperatively, small perforations noted at the descending colon just proximal to anastomosis area, with generalised contamination of peritoneal cavity with turbid contrast fluid.

A 50-year-old man with no medical history was diagnosed as having low rectal cancer (T2N1M0) and underwent ULAR with covering ileostomy after neoadjuvant chemo-radiation. One year later, he underwent a routine distal loopogram examination for the assessment of anastomosis patency. The distal loopogram was performed using water-based contrast prior to closure of ileostomy. The procedure which was done as an outpatient procedure showed a colonic stricture measuring five centimetres in length, proximal to the coloanal anastomosis. However, there was no evidence of anastomotic contrast leakage noted on this study. He was discharged home after the procedure. Eight hours later, the patient returned to the emergency department with generalized peritonitis and septic shock (blood pressure 86/56mmHg, pulse rate 130bpm, temperature 37.8 degree Celsius). A plain abdominal radiograph showed extra-luminal contrast (Figure 4), which was confirmed by CT abdomen (Figure 5). He was resuscitated with fluids and started on broad-spectrum antibiotics. Emergency laparotomy was performed the same day. Intra-operatively, two litres of foul-smelling, turbid, whitish fluid were found in the abdominal cavity. There were three small perforations noted at the descending colon just proximal to the anastomotic stricture (Figure 6). Peritoneal lavage and primary closure of the perforations were done. A drain was placed in the pelvis adjacent to the repair site. Post-operatively, the patient was continued on broad-spectrum antibiotics, and his condition improved gradually. The drain was removed on post-operative day 6. The patient was discharged well two weeks after the emergency operation but never had the ileostomy closed as he refused further surgery.

Ethical approval: The research related to human use has been complied with all the relevant national regulations, institutional policies and in accordance the tenets of the Helsinki Declaration, and has been approved by the authors’ institutional review board or equivalent committee.

Informed consent: Informed consent has been obtained from all individuals included in this study.

## 3 Discussion

Contrast studies, like distal loopograms and proctograms, are routinely requested by many colorectal surgeons to assess anastomoses before the closure of covering ileostomies. This is usually done in addition to clinical assessment by digital rectal examination and endoscopy.

However, there are arguments about the necessity of routine contrast studies in this respect, as there is doubt about the ability of contrast studies to predict anastomotic complications [2, 3].

Generally, contrast studies of the gastrointestinal (GI) tract are considered safe, with reported perforation rates between 0.02 and 0.04% [5], and thus, can be performed as outpatient procedures. There are different contrast agents to choose from, and these are mainly divided into water-soluble (e.g. Gastrografin) and water-insoluble (e.g. barium). Water-soluble contrast is the contrast of choice when perforation of the GI tract is suspected because it is thought to cause less peritoneal irritation than barium. Nevertheless, it still has significant potential to cause peritonitis, as demonstrated in our cases. There is a paucity of literature on the subject, as the incidence is so low. In a Korean series of 141 patients who had contrast enema study before closure of ileostomy, only one patient had iatrogenic bowel perforation during the barium enema examination and developed barium peritonitis [2]. The main difference in our series is that water-soluble contrast agent was used in both of our patients instead of barium enema.

In addition, it was interesting to note that both of our patients did not present with any immediate symptoms during the distal loopogram procedure. As outpatients, both patients were discharged home soon after the procedures. The first case presented to the emergency department with abdominal pain two days after; whereas the second case return to the hospital 8 hours after discharge. From our literature search, ours are probably the first case reports showing delayed bowel perforation leading to overt peritonitis after distal loopogram with water-soluble contrast agent. In contrast to our cases, which both presented as delayed bowel perforation, two earlier cases reported bowel perforation with barium peritonitis that happened within minutes after the injection of barium contrast [6, 8].

In terms of the mechanism of perforation, some have reported perforation at the catheter insertion site and attributed the perforation to local trauma by the contrast injecting catheter itself [6]. This was the most likely cause for our first patient, as the perforation site was located near to the loop ileostomy. The perforation of the ileum probably occurred during the multiple failed attempts at channeling the contrast injecting catheter and might be compounded by forceful injection of contrast agent. This emphasizes that the insertion of the catheter through the correct ileostomy lumen might not be straightforward, especially to staff who are not familiar with the anatomy of the loop stoma. Advice from the primary surgical team should be sought if the anatomy of the stoma was not clear-cut.

Gastrografin is a salt of the amidotrizoic acid, and when introduced into the bowel lumen, it draws fluid from the plasma and interstitium due to osmotic gradient [7]. The fluid shift into a closed compartment such as an obstructed colon could lead to over-distention of the bowel. Moreover, prolonged retention of Gastrografin in the gastrointestinal tract (e.g. stasis) may carry the risk of tissue damage, bleeding, and bowel necrosis [7]. This risk is compounded if the distended bowel is the descending colon, which is usually smaller in calibre, and less distensible compared to the rectum or sigmoid colon [8]. This phenomenon probably explains the perforation in the second patient, especially if the contrast was injected under high pressure.

There have not been many publications related to the further management of patients after contrast peritonitis. This may be partly because of the low incidence rate. Nonetheless, dense bowel adhesions and ureteric obstruction are recognized late complications of barium peritonitis [5], and the management is complex. On the other hand, Gastrografin, being water-soluble, is absorbed and excreted mainly by the kidneys [9]. Early recognition and surgical management usually lead to satisfactory outcomes. In both of these patients, the surgeon did not encounter much bowel adhesions during the surgery, as there was not much delay in surgical management. Furthermore, the usage of Gastrografin instead of barium might have led to lesser bowel adhesions encountered during the surgery.

In conclusion, although distal loopogram is deemed to be a safe procedure especially when a water-soluble contrast agent is used, these two cases highlight its potential danger. The practice of routine distal loopogram before the closure of ileostomy should be reconsidered, especially when clinical examination may offer a safer alternative to most cases. If distal loopogram is deemed really necessary, precautions should be taken, and patients should be properly counselled prior to the procedure. Patients need to be aware of the possibility of delayed bowel perforation that may lead to late presentation.
